# Cephalalgiaphobia as a feature of high-frequency migraine: a pilot study

**DOI:** 10.1186/1129-2377-14-49

**Published:** 2013-06-10

**Authors:** Giulia Giannini, Stefano Zanigni, Daniela Grimaldi, Roberto Melotti, Giulia Pierangeli, Pietro Cortelli, Sabina Cevoli

**Affiliations:** 1 IRCCS Istituto di Scienze Neurologiche, Dipartimento di Scienze Biomediche e NeuroMotorie, Alma Mater Studiorum University of Bologna, Bologna, Italy; 2 Center for Biomedicine, European Academy Bozen/Bolzano (EURAC), Affiliated institute of the University of Lübeck, Bozen/Bolzano, Italy; 3 Sleep, Metabolism and Health Center, Department of Medicine, The University of Chicago, Chicago, IL, USA

**Keywords:** Migraine, Cephalalgiaphobia, Anxiety disorders, Chronic migraine

## Abstract

**Background:**

Cephalalgiaphobia is the fear of having a headache attack during a pain-free period that may induce patients to use analgesic in the absence of pain to prevent headache and to improve their performances. This study aims at assessing if cephalalgiaphobia is related to migraine frequency or medication overuse, and if it is per se a predictor of increase in migraine frequency.

**Methods:**

This is a pilot prospective cohort study on 126 consecutive migraineurs referred to a tertiary Headache Centre. A headache specialist collected data regarding migraine features, frequency and medications at baseline (T0) and 2 years later (T1). Cephalalgiaphobia was investigated at T0 and T1 through a score determined by a 4 items questionnaire.

**Results:**

Moderate-high migraine frequency was associated with higher risk of cephalalgiaphobia (p < 0.001). Chronic migraineurs with medication overuse had higher score of cephalalgiaphobia than those without medication overuse (p < 0.001). Patients with increased migraine frequency between T0 and T1 had higher cephalalgiaphobia score (p < 0.001).

**Conclusions:**

Cephalalgiaphobia may represent a high-frequency migraine feature and may play a role in chronicization. Therefore, it should be better investigated by clinicians and treated or prevented in order to reduce the risk of disability and the increase in migraine frequency.

## Background

The relationship between migraine and some psychological features has been reported for more than a century. A strong bidirectional association between migraine and depression or anxiety has been demonstrated by several studies while the connection with eating, bipolar and other disorders still remains possible [1-6]. An increased risk of suicide attempt has been demonstrated in migraineurs, especially in those suffering from migraine with aura [1-7]. Moreover, patients suffering from migraine and chronic daily headache reported severe hopelessness and perceived disability [6]. This comorbidity increases therefore the severity of perception of headache symptoms and impact the quality of life, posing additional challenges for an effective patient management. Psychiatric disorders can also modify the migraine course raising the risk of transformation in a chronic form [1,2,6]. The link between migraine and lifetime anxiety disorders (panic, obsessive-compulsive disorders, generalized anxiety, phobias) was described in both clinical and population-based studies [1-6,8-12]. It has been demonstrated that the prevalence of anxiety-spectrum disorders in migraineurs ranges from 9.1% to 24.6%, compared to 2.5-12.0% in general population [8,13,14]. A phobia is a persistent, excessive, and unreasonable fear of a specific object or situation that induces to avoid it, despite the awareness and reassurance that it is not dangerous. It can significantly interfere with social and occupational functioning [15]. A few studies focused on the relationship between migraine and phobic disorders: Ratcliffe and colleagues [14] investigated the prevalence of mental disorders in migraineurs compared to healthy controls showing an increased rate of social phobia [3.0% vs. 1.9%, odds ratio (OR) = 1.38], agoraphobia (6.3% vs. 1.5%, OR = 3.21) and simple phobia (15.1% vs. 6.8%, OR = 1.97). Conversely, Senaratne et al. [16] found a migraine prevalence of 22.0% in patients suffering from phobia. Merikangas et al. [17], in a prospective study, registered an increased risk of phobia, especially of simple and social phobia, in migraineurs with aura. In a prospective study of the Epidemiologic Catchment Area Study of Baltimore [18], Swartz et al. highlighted how phobia was predictive of incident migraine [OR = 1.70, 95% confidence interval (CI): 1.11-2.58]. Moreover, authors reported also a significant association between prevalent migraine and phobia (OR = 1.43, 95% CI: 1.07-1.91), in particular agoraphobia (OR = 1.88, 95% CI: 1.33-2.67) and simple phobia (OR = 1.35, 95% CI: 1.02-1.79) [18]. Although psychiatric disorders are not a comorbidity of migraine only, they may play an important role in its chronicization as well as in medication overuse [1,5,19-27]. Corchs et al. [28] investigated the psychiatric profile of patients suffering from chronic migraine showing that 60.7% of them presented a phobic anxious condition in their lifespan, 35.7% had a diagnosis of specific phobia, 26.8% had social phobia and two agoraphobia. Comparing migraineurs and patients suffering from Medication Overuse Headache deriving from migraine, Radat et al. [24] registered an increased risk of social phobia (34.1% vs. 12.2%; OR = 4.3, 95% CI: 1.3 – 14.5) in the second group. A specific kind of phobia directed against illness has been also described and distinguished from hypochondria [29]. In this condition, patients who have experienced a health problem develop the fear that it might reappear again: that is what happens for example in patients with heart diseases or other chronic conditions and also in migraineurs, scared to have a major attack of migraine. This last trouble, called cephalalgiaphobia, has been described by Peres et al. [29] as a new possible specific illness phobia. In their study, they investigated a sample of 12 patients followed at a tertiary headache clinic in Brazil showing anticipatory anxiety, fear of a headache attack during a pain-free period or worsening of the pain during a period of mild headache. As avoidance behavior, patients overused acute medications despite being aware that it was the fear of having another migraine attack or a headache exacerbation that made them take more analgesics than necessary. Authors hypothesized also that cephalalgiaphobia may decrease the threshold for initiating the analgesic consumption behavior leading to acute medication overuse [29]. No other study on this topic has been published to date. Our study aims at evaluating whether cephalalgiaphobia was related to migraine frequency, aura status, medication overuse and, finally, if it was per se a predictor of increase in migraine frequency.

## Methods

This is a prospective pilot study carried out on patients consecutively referred between November 2009 and December 2009 to the tertiary headache center of the Neurological Department, University of Bologna, Northern Italy. The institutional review board of the Department of Neurological Sciences of the University of Bologna approved the project and all participants signed informed consent at baseline. Patients aged < 18 years and suffering from Tension-type Headache, Cluster Headache or secondary headaches were excluded. Migraine was defined using ICHD-II criteria [30] while Medication Overuse was defined using the ICHD-IIR criteria [31]. Cephalalgiaphobia was investigated by means of a non-validated structured interview (4 items) at baseline (T0) and at follow-up (T1) and a score ranging from 0 to 8 was determined according to the frequency (never = 0; sometimes = 1; often/always =2) of the following events:

1. When you are feeling well do you ever fear to have a migraine attack?

2. Have you ever used painkillers even though you were not having pain just because you were scared of a possible migraine attack?

3. Have you ever used a second dose of painkilling drugs just because you feared that the pain would get worse before it actually did?

4. Have you ever used painkillers to improve your performances and be more active, although you were not feeling the pain at all?

This structured interview was formulated on the basis of our clinical expertise taking also into account specific phobia criteria described on DSM-IV [15]. At the time of the first visit (T0) a headache specialist performed a semi-structured interview indicating the diagnosis and the frequency of headache over the last 3 months with a headache diary. Information about acute migraine treatments was collected in all subjects. Patients were recalled 2 years later (T1), from December 2011 to January 2012, by the researchers who performed a telephone-interview during which they administered the cephalalgiaphobia questionnaire and reported any changes in headache frequency (self-reported by patients on the basis of their diaries) and the monthly amount of medications for acute migraine treatment. We compared the cephalalgiaphobia score at T0: a) among groups with a different frequency of migraine (1-3 attacks per month; 4-14 attacks per month and ≥ 15 attacks per month); b) between episodic migraineurs with aura and those without aura; c) between patients suffering from chronic migraine with medication overuse and those without medication overuse. We also examined if the cephalalgiaphobia could be a predictor of worsening of migraine frequency comparing the score among patients who improved, remained in the same group or worsened at T1 compared to T0. Kruskal-Wallis Test was performed to compare variables as the cephalalgiaphobia score with an asymmetrical (non-normal) distribution. A multiple test adjusting procedure, based on the rank-means paired differences, has been applied if the overall test was significant at the 0.05 level [32]. The relationship between cephalalgiaphobia and migraine was further investigated both at T0 and T1 and between time points to assess their reciprocal influence. Scores of cephalalgiaphobia were grouped into 4 categories (0, 1, 2, ≥3) due to sparse extreme cell counts. Crossed-time periods analysis used an ordered logistic regression to assess the possible influence of initial values of cephalalgiaphobia on migraine at T1, accounting for initial frequency. The odds ratio from the regression model can be interpreted as the factor by which the odds of scoring one level higher in the outcome (cephalalgiaphobia) increases for each additional level of the exposure of interest (frequency of migraine). Significance level was set at p ≤ 0.05. Data analysis was performed with STATA® version 12.0.

## Results

### Sample analysis

At baseline 126 (24 males and 102 females) patients were included in this study. At follow-up (T1), 124 patients (23 males and 101 females) were recalled by researchers while two patients were lost because telephonically unreachable (Table 1). Sample characteristics at T0 and T1 regarding migraine frequency, aura status and medication overuse are reported in Table 1. As for patients whose migraine frequency changed compared to baseline, 76 patients remained on the same level (17 reported 1-3 attacks per month, 40 4-14 attacks per month and 19 ≥ 15 attacks per month), 33 patients improved (of 15 patients suffering from chronic headache at T0, 11 reported 4-14 attacks per month and four 1-3 attacks per month at T1; 18 patients suffering from 4-14 attacks per month at T0 reported 1-3 attacks per month at T1) and 17 patients worsened (9 patients with 4-14 attacks per month turned to chronic headache at T1; of 8 patients with 1-3 attacks per month at baseline, 6 reported 4-14 attacks per month and 2 ≥15 attacks per month at T1). The cephalalgiaphobia mean score at T0 was 1.56 [median (med): 1, interquartile ranges (iqr): 1-2] and at T1 was 1.57 (med: 1, iqr: 0-2) (Table 1).

**Table 1:** Headache characteristic of sample

		**T0**	**T1**
**Sample**	TOT	126	124
	Males	24 (19.05%)	23 (18.55%)
	Females	102 (80.95%)	101 (81.45%)
	Mean age±SD	41.41 ± 12.72	43.76 ± 12.19
**Migraine’s frequency**	1-3 attacks/month	25 (19.84%)	39 (31.45%)
	4-14 attacks/month	66 (52.38%)	56 (45.16%)
	≥15 attacks/month	35 (27.78%)	29 (23.39%)
**Aura status**	migraine with aura	10 (10.99%)	10 (11.58%)
	migraine without aura	81 (89.01%)	84 (88.42%)
**Chronic migraine**	with MO	18 (51.43%)	21 (72.41%)
	without MO	15 (42.86%)	8 (27.59%)
	missing data	2	――
**Cephalalgiaphobia Score**	(mean/Med/Iqr)	1.56/1/1-2	1.57/1/0-2

**Legend**: *Iqr* Interquartile Ranges, *Med* Median, *MO* Medication overuse, *SD* Standard Deviation.

### The cephalalgiaphobia score and the characteristics of migraine

**Figure 1:**
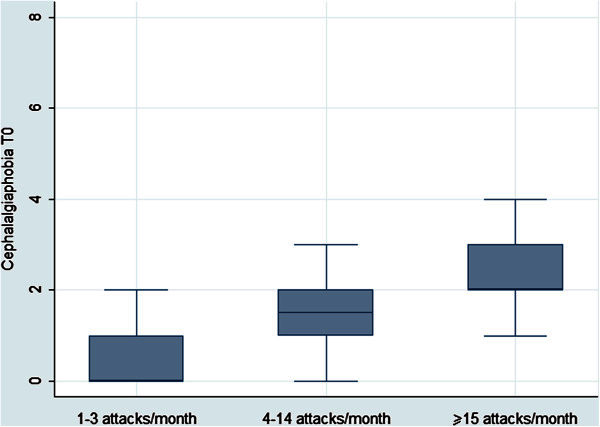
Cephalalgiaphobia and frequency of migraine.

When comparing the cephalalgiaphobia score among groups with a different frequency of migraine (1^st^ group = 1-3 attacks per month; 2^nd^ group = 4-14 attacks per month and 3^rd^ group ≥ 15 attacks per month) our study showed that the more frequent the migraine, the higher was the cephalalgiaphobia score. Patients suffering from chronic headache had an increased score of cephalalgiaphobia compared to those suffering from 4-14 or 1-3 attacks per month [mean, med (iqr): 2.48, 2 (2-3) vs. 1.77, 1.5 (1-2) vs. 0.69, 0 (0-1)] (Figure 1). The Kruskal-Wallis test revealed statistically significant differences among groups (p < 0.001). A multiple test adjusting procedure, based on the rank-means paired differences, showed that this statistical significance was attributable to the difference between 2^nd^ vs. 1^st^ group (p < 0.001) and 3^rd^ vs. 1^st^ (p < 0.001) but not to 3^rd^ vs. 2^nd^ group (Table 2). As for the aura status we did not register a difference between episodic migraineurs suffering from migraine with aura [mean: 1.31, med (iqr): 1 (0-2)] and migraineurs without aura [1.60, 1.5 (0-2)] (p = 0.72) (Table 2). In exploring the relation between the cephalalgiaphobia and the medication overuse, our study showed that patients overusing drugs displayed an increased score of phobia [mean: 2.91, med (iqr): 2 (2-4)] compared to those not overusing them [mean: 1.38, med (iqr): 1.5 (0.5-2)]. The Kruskal-Wallis test revealed statistically significant differences among groups (p = 0.029), also confirmed by a multiple test adjusting procedure based on the rank-means paired differences (p = 0.018) (Table 2).

**Table 2:** The cephalalgiaphobia score and the characteristic of migraine at T0

		**Mean**	**Med**	**Iqr**	**p**	**p Adj**
**Migraine’s frequency**	1-3 attacks/month	0.69	0	0-1	**0.0001**	1-3 vs. 4-14 = **0.000092**
	4-14 attacks/month	1.77	1.5	1-2		4-14 vs. ≥ 15 = 0.037049 (NS)
	≥ 15 attacks/month	2.48	2	2-3		≥ 15 vs. 1-3 = **0.000001**
**Aura status**	migraine with aura	1.31	1	0-2	0.717 (NS)	――
	migraine without aura	1.60	1.5	0-2		
**Chronic migraine**	with MO	2.91	2	2-4	**0.02891**	**0.017944**
	without MO	1.38	1.5	0.5-2		

**Legend**: *Adj* Adjusting p-value for multiple comparisons, *Iqr* Interquartile Ranges, *Med* Median, *MO* Medication overuse, *NS* statistically not significant.

### Cephalalgiaphobia as a predictor of worsening in migraine frequency

Comparing T0 to T1, patients whose migraine frequency worsened [mean, med (iqr): 2.47, 2 (1-3)] had an increase cephalalgiaphobia score compared to those who remained on the same level [1.45, 1 (1-2)] or those who improved [1.36, 1 (1-2)]. The Kruskal-Wallis test revealed statistically significant differences among groups (p = 0.038). A multiple test adjusting procedure, based on the rank-means paired differences, showed statistical significance both in patients who worsened vs. those who remained on the same level (p = 0.011) but also vs. those who improved (p = 0.012). The ordered logistic regression to assess the possible influence of initial values of cephalalgiaphobia on migraine at T1, accounting for the initial frequency, showed an OR =1.53 (CI: 0.94-2.50), p = 0.09.

## Discussion

To our knowledge, this is the first prospective study investigating cephalalgiaphobia in a migraine cohort. Our results showed that migraineurs with higher attacks frequency suffered more frequently from cephalalgiaphobia, fearing new attacks and taking therefore more easily attack drugs. Cephalalgiaphobia was also more frequent in patients with medication overuse compared to those without it. Cephalalgiaphobia scores resulted lower in patients with a low frequency of migraine attacks (1-3/month) if compared to those with moderate-high frequency (4-14/month) or chronic migraine (≥15/month). There was no difference between moderate-high frequency and chronic patients, probably because the first group was a large range, including patients with a very high frequency of attacks, near to chronic migraine. Our results also highlighted that cephalalgiaphobia could play a role in the worsening of migraine frequency. In fact, patients whose migraine frequency worsened had an increase in cephalalgiaphobia score at follow-up compared to those who remained on the same level or those who improved. The possible association between migraine worsening and cephalalgiaphobia could be explained in a direct and an indirect way. The first hypothesis considers that cephalalgiaphobia, as other phobias, directly increases migraine frequency. In the indirect hypothesis, migraine worsening could be provoked by an increase in medication intake, a well-known risk factor for chronicization, due to cephalalgiaphobia. We therefore agree with Peres and colleagues [29] who hypothesized that cephalalgiaphobia may decrease the threshold for initiating the analgesic consumption behavior, leading to acute medication overuse. Our study presents though many limitations. The major limit was that cephalalgiaphobia was investigated through a non-validated questionnaire, since an objective definition of cephalalgiaphobia is not available to date. Moreover, possible confounders such as psychiatric comorbidity and concomitant medications were not systematically collected and therefore not taken into account. Other limits were represented by the small sample size and the tertiary care setting of the study that may have increased the proportion of subjects with more severe headache and consequently biased the selection of patients. Lastly, in our study, quality of life was not assessed using standardized questionnaires and for this reason it could not be used as an outcome in the analysis.

## Conclusions

Cephalalgiaphobia is a trouble that needs to be commonly recognized with its own specific clinical definition. Despite the limits of this study, our results suggest that cephalalgiaphobia should be better investigated by clinicians and, if present, it should be better treated in order to reduce the risk of disability and migraine frequency increase. A network of psychiatric clinics and psychologists should be developed and involved for patients suffering from cephalalgiaphobia in order to treat this disorder either pharmacologically or through psychological techniques, for example self-help group [33]. Further studies are necessary in order to investigate extensively the relationship in terms of causality between cephalalgiaphobia and increase in migraine frequency. It is mandatory to consider also other well-known risk factors for chronicization, such as psychiatric illness and life events, and other possible modifying features, such as concomitant medication and treatment adherence.

## Competing interest

Prof. Pietro Cortelli has received honoraria for speaking engagements or consulting activities with Allergan Italia, Boehringer Ingelheim Italia, Chelsea Therapeutics, GlaxoSmithKline S.p.A , Lundbeck Italy, Merck Sharp & Dohme (Italia) , Teva, UCB Pharma S.p.A, Chiesi Farmaceutici, AbbVie srl. The other authors declare that there is no conflict of interest.

## Authors’ contributions

GG: acquisition, analysis and interpretation of data, drafting the manuscript. SZ: analysis and interpretation of data, drafting the manuscript. DG: acquisition of data, drafting the manuscript. RM: analysis and interpretation of data. GP: substantial contributions to conception and design, acquisition of data, revising manuscript critically for important intellectual content. PC: substantial contributions to conception and design, revising manuscript critically for important intellectual content. SC: substantial contributions to conception and design, acquisition and interpretation of data, revising manuscript critically for important intellectual content. All authors read and approved the final manuscript.
